# Is Covid-19 lockdown related to an increase of accesses for seizures in the emergency department? An observational analysis of a paediatric cohort in the Southern Italy

**DOI:** 10.1007/s10072-020-04824-5

**Published:** 2020-10-23

**Authors:** Federica Palladino, Eugenio Merolla, Marella Solimeno, Maria Fulvia de Leva, Selvaggia Lenta, Onorina Di Mita, Anna Bonadies, Pasquale Striano, Vincenzo Tipo, Antonio Varone

**Affiliations:** 1 Department of Women’s and Children’s Health and General and Specialized Surgery, University of Campania “Luigi Vanvitelli”, Via Luigi de Crecchio 2, 80138 Naples, Italy; 2 Clinic of Child and Adolescent Neuropsychiatry, Department of Mental Health, Physical and Preventive Medicine, University of Campania “Luigi Vanvitelli”, Via Pansini 5, 80131 Naples, Italy; 3 Pediatric Neurology, Department of Neuroscience, Santobono-Pausilipon Children’s Hospital, Via Mario Fiore 6, 80129 Naples, Italy; 4 Pediatric Emergency Department, Santobono-Pausilipon Children’s Hospital, Via Mario Fiore 6, 80129 Naples, Italy; 5 Pediatric Neurology and Muscular Diseases Unit, “IRCCS Istituto Giannina Gaslini”, Genova, Italy; 6 Department of Neurosciences, Rehabilitation, Ophthalmology, Genetics, Maternal and Child Health, University of Genova, Genova, Italy

**Keywords:** Seizures, Lockdown, Mobile media devices, Sleep disorder, Children, COVID-19

## Abstract

**Background:**

The World Health Organization (WHO) declared a global pandemic of Covid-19 on 11 March 2020. The lockdown caused a lifestyle changes: an increase in the use of mobile media devices (MMDs), sleep and psychiatric disorders, incorrect habits regarding food and physical activities.

We investigate prevalence of admission for seizures at our emergency department (ED), during Italian lockdown, comparing with that of the same period of the previous year (2019), and the relationship with some lifestyle changes.

**Methods:**

In this observational study, patients (4–14 years) with seizures that accessed at our ED, during Italian lockdown, were eligible. Non-epileptic events and febrile seizures were excluded. We describe two groups: patients with new-onset seizures and not. Moreover, a questionnaire concerning use of MMDs and sleep habits was administered.

**Results:**

Fifty-seven patients were included; median age 8.03 years. Considering only paediatric medical emergencies, the prevalence of accesses for seizures was 2.6% (CI 95% 0.020–0.034), while the incidence was 0.94% (CI 95% 0.006–0.0149). There was a statistically significant difference with prevalence of previous years, χ^2^ 102.21 (*p* = 0.0001). We also reported a difference in daily screen time (DST) (*p* = 0.001) and total sleep time (TST) (*p* = 0.045), in all population, between period pre- and during lockdown. A negative correlation between DST and seizures latency (*Spearman’s ρ* -0.426, *p* = 0.038) was found. In the two groups, the results were partially overlapping.

**Conclusions:**

During lockdown period, we assisted to an increase of accesses for seizures. It is conceivable that a sleep time change and/or higher MMD use could act as triggers for seizures.

**Electronic supplementary material:**

The online version of this article (10.1007/s10072-020-04824-5) contains supplementary material, which is available to authorized users.

## Background

The World Health Organization (WHO) declared a global pandemic of Covid 19—the disease caused by Sars–Cov2—on Wednesday 11 March 2020 (https://www.who.int/dg/speeches/detail/who-director-general-s-opening-remarks-at-the-media-briefing-on-covid-19%2D%2D-11-march-2020), first reported in Wuhan (China) in December 2019 [1].

Since then, according to literature [2, 3], we have witnessed a worldwide massive drop in emergency department (ED) paediatric admissions, partially due to both the higher prevalence of this disease in older age groups [4] and mild-asymptomatic clinical course in children and adolescents [5].

This phenomenon is to relate, as well, to a reduction of other seasonal viral infections due to self-isolation and social distancing measures adopted by governments. Nevertheless, parental concerns about exposing their children to hospital environment delayed diagnosis of acute severe illnesses that needed to be treated [6].

The lockdown, isolation, contact restrictions and economic shutdown caused a change of the psychosocial environment. As a concomitant consequence, an increase of mental health disorders, in the paediatric population, was observed (anxiety, depression, panic attacks) [7].

Children and adolescents were placed under pressure by this new situation that included smart schooling, limited access to outdoor play spaces and sleep disorders [8]. As a consequence, the use of mobile media devices (MMDs), television, smartphones, tablets, computers and video consoles (PlayStation™, Xbox™, Nintendo™) was increased.

We aimed to correlate lifestyle changes during the Covid-19 pandemic with the occurrence of epileptic seizures in a paediatric population from Southern Italy. Moreover, we examined the correlation between seizures, increasing usage of MMDs and sleep disorders, during lockdown.

## Materials and methods

This observational study involved patients attending ED of Santobono-Pausilipon Children’s Hospital, one of the major paediatric hospitals in Southern Italy, during the Covid-19-related lockdown in Italy (since the 9th of March 2020 up to 4th of May 2020). In this study, all patients entering the ED for seizures, from 4 to 14 years of age, were included. Non-epileptic events and febrile seizures were excluded. Our cohort was stratified into two groups, i.e. patients presenting with new-onset seizures and patients with a previous diagnosis of epilepsy.

Data including demographic characteristics (gender, age, family history for seizures), seizures semiology, previous diagnosis of epilepsy, pharmacological treatment and compliance to therapy before the seizure, admission at the neurological department or not, were collected. In addition, neurological exam, electroencephalographic (electroencephalography (EEG)) and neuro-radiological (computerized tomography CT or magnetic resonance imaging MRI) findings and diagnosis of epilepsy at discharge were reported. These data were collected analysing the ED database and medical records of the patients admitted to our Neurology Department.

Moreover, a brief questionnaire (appendix 1, English translation of the Italian administered version) was created to obtain data about the use of MMDs (daily screen time (DST); typology of device, television, smartphones, tablets, computers and video consoles and seizures latency from the last use of the device), sleep habits (total sleep time (TST), qualitative alterations of sleep) and how the usage varied in the lockdown period comparing it to the habitual mean usage. The questionnaire is composed of eleven questions, elaborated by adapting other existing and/or validated questionnaires [9–11]. It was administered via telephone to one of the child’s parent; a verbal consent was obtained before the administration.

To compare the 2020 prevalence of admission for seizures at our ED with that of the previous year, we also reported the number of patient (4–14 years) attending our ED for seizures, from March up to April 2019. Furthermore, data including demographic characteristics (gender, age) and diagnosis of discharge were recorded.

Regarding discharge diagnosis, we reported the number of patients with final epilepsy diagnosis or only single seizures, and we described them according to the new classification of seizures and epilepsy of International League Against Epilepsy (ILAE) of 2017 [12].

### Statistical analysis

Prevalence and incidence of admissions for seizures at our ED, during Italian lockdown period (9th March to 4th May 2020) and during the same period of 2019, were described. We compared prevalence and incidence of seizures at our ED of 2020 with those of 2019, respectively, using chi-squared test.

Continuous non-parametric variables are presented as median, IQR and range, whereas categorical variables are expressed as number and percentage. Wilcoxon test and Mann-Whitney U test were used to compare continuous non-parametric variables. Chi-squared test and Fisher’s exact test were used for testing relationships between categorical variables. Spearman’s correlation was used to evaluate the association between DST and age, DST and seizure latency; in case of a significant correlation, a simple linear regression was performed. Exclusively for patients with a discharge diagnosis of focal and generalized epilepsy, Binary logistic regression model was used to explore predictors for these diagnoses (DST, number of devices, TST). For all analyses, *p* values < 0.05 were considered statistically significant. IBM SPSS Statistics 22 Software for Windows was used for statistical analysis.

## Results

From the 9th of March up to the 4th of May 2020, there were 3968 total accesses to our ED, 61 of them (1.5%, CI 95% 0.012–0.019) for critical events, 57 (1.4%, CI 95% 0.011–0.018) for seizures and 4 (0.1%, CI 95% 0.0005–0.0034) for non-epileptic paroxysmal events. Incidence of seizures was 0.5% (CI 95% 0.003–0.008) (20/3931). Excluding surgical (829) and orthopaedic (553) accesses, and non-urgent (426) patients, the prevalence of seizures was 2.6% (57/2160) with CI 95% 0.020–0.034. Incidence of seizures was 0.94% (CI 95% 0.006-0.014)(20/2123), while the incidence of new epilepsy’s diagnosis was 1.3% (CI 95% 0.009–0.018)(28/2131). In the same period, last year, we recorded 16,923 admissions in ED, 40 (0.24%) for critical events (1/40 patients had non-epileptic paroxysmal event). Prevalence of seizures was 0.23% (CI 95% 0.001–0.003) (39/16923), and the incidence was 0.08% (CI 95% 0.0004–0.001) (13/16886). Excluding surgical (2424) and orthopaedic (2319) accesses, and non-urgent (2397) patients, the prevalence and incidence of seizures were 0.4% (CI 95% 0.003–0.005) (39/9783) and 0.13% (CI 95% 0.0008–0.0023) (13/9757), respectively. Incidence of new epilepsy’s diagnosis was not reported because no reliable data were available. There was a statistically significant difference between the prevalence and incidence of seizure accesses between March and May 2020 and 2019: about prevalence χ^2^ = 102.21 (*p* = 0.0001), while incidence χ^2^ 37.34 (*p* = 0.0001), in comparison to all accesses, whilst considering only the paediatric medical emergencies χ^2^=111.36 (*p* = 0.0001) for prevalence and χ^2^=41.18 (*p*= 0.0001) for incidence.

There were not statistically significant differences about patients with new-onset seizures and known epilepsy. Data were reported in Table 1.

**Table 1:** Admissions at emergency department. Categorical variables are expressed as a number and percentage

Admissions at emergency department	Study population		*P* value
	*2020*	*2019*	
All accessess to the ED	3968	16923	
o *Surgical patients*	829	2424	
o *Orthopedic patients*	553	2319	
o *Non-urgent*	426	2397	
*o Medical emergencies*	2160	9783	
o *Seizures*	57	39	
*New-onset seizures*	*20*	*13*	*0.8589*
*Known epilepsy or not new-onset seizures*	*37*	*26*	
o *Non-epileptic events*	4	1	
Prevalence of seizures	2.6 %	0.23 %	*0.00001*
Incidence of seizures	0.94%	0.13%	*0.00001*
Incidence of new epilepsy’s diagnosis	12.96 %	-	

## Study Population

Fifty-seven patients were enrolled in the study, 20 of them had new-onset seizures (35 %), whilst 37 (65%) had known epilepsy or not new-onset seizures. Clinical and demographic data of both groups were summarised in Table 2.

**Table 2:** Demographic and clinical characteristics of two groups: patients with new-onset seizures and patients with known epilepsy and not new-onset seizures, of 2020 and 2019 year. Categorical variables are expressed as a number and percentage. Continuous non-parametric variables are reported as the median, range and interquartile [IQR]

Demographic and clinical characteristics	2020	2019
Patients with new-onset seizures		
N of patients	20	13
Gender (male)	9 (45%)	8 (61.5%)
Age (years)	8(4–12) [4.25–9.75]	6 (4–10)[5-8]
Familiarity for seizures		
None	11 (55%)	n.a.
Epilepsy	8 (40%)	
Febrile seizures	1 (5%)	
Hospitalised patients	17 (85%)	3 (23%)
*Admission diagnosis*		
Seizures onset	20	13
*Focal*	7 (35%)	-
*Generalized*	9 (45%)	1 (7.7%)
*Unknown*	4 (2%)	12 (92.3%)
*Discharge diagnosis*		
Epilepsy	17 (85%)	3 (23%)
*Focal*	11 (64.7%)	2 (66.7)
*Generalized*	4 (23.5%)	1 (33.3%)
*Combined focal and generalized*	1 (5.9%)	-
*Unknown*	1 (5.9%)	-
Seizures onset	3 (15%)	10 (77%)
*Focal*	1 (33.3%)	1 (10%)
*Generalized*	2 (66.7%)	-
*Unknown*	-	9 (90%)
Patients with known epilepsy and not new-onset seizures		
N of patients	37 (65%)	26 ( 66.7%)
Gender (male)	16 (43.2%)	16 (61.5%)
Age (years)	8 (4-14) [6-12]	9.5 (5-13) [6.23-12]
Familiarity		
None	22 (59%)	n.a.
Epilepsy	11 (30%)	
Febrile seizures	4 (11%)	
Hospitalised patients	20 (54%)	3 (11.5%)
*Admission diagnosis*		
Seizures onset	13	8
*Focal*	4 (31%)	-
*Generalized*	3 (23%)	-
*Unknown*	6 (46%)	8
Epilepsy	24	18
*Focal*	14 (58.3%)	15 (83.3%)
*Generalized*	7 (29.2%)	3 (16.7%)
*Combined focal and generaized*	-	-
*Unknown*	3 (12.5%)	-
*Discharge diagnosis*		
Epilepsy	35	21
*Focal*	19 (54.3%)	17 (81%)
*Generalized*	11 (31.4%)	3 (14.3%)
*Combined focal and generaized*	2 (5.7%)	-
*Unknown*	3 (8.6%)	1 (4.7%)
Seizures onset	2	5
*Focal*	-	-
*Generalized*	-	.
*Unknown*	2	5

### Patients with new-onset seizures

Of 20 patients, 9 (45%) were male and 11 (55%) female. The median age was 8 years (range 4.00–12.00) [IRQ 4.25–9.75]. Eight patients (40%) had familiarity for epilepsy; one patient (5%) for febrile seizures and 11 (55%) had not familiarity for epilepsy and/or seizures. In 17 patients (85%), neurological clinical examination was normal. Seventeen of 20 patients were hospitalized (85%).

At admission, 7 of 20 (35%) patients had focal onset seizures (FOS), 9 (45%) had generalized onset seizures (GOS) and 4 (2%) had unknown onset seizures (UOS). Nineteen of 20 performed an EEG that showed a pathological pattern in 16 (80%) patients: generalized epileptic anomalies in 3 patients and focal anomalies in 13 patients (9 temporal, 3 frontal and 1 multifocal). Only one patient (5.3%), 8 years old, was photosensitive.

Nineteen of 20 patients had undergone CT (16 patients) or MRI (3 patients) at least once in their life. In detail, for 2 patients, MRI showed a structural aetiology (one had a brain tumour and the other had a malformation of cortical development, polymicrogyria).

Discharge diagnoses were epilepsy for 17 of 20 patients (85%): 11 (64.7%) were focal epilepsy (FE); 4 (23.5%) were generalized epilepsy (GE); 1 (5.9%) were combined focal and generalized epilepsy (CFGE); and 1 (5.9%) were unknown epilepsy (UE). On the other hand, 2 of 20 patients (15%) were diagnosed with seizures onset type: 1 (33.3%) had focal onset seizure (FOS), and 2 (66.7%) had generalized onset seizure (GOS).

Regarding epilepsy aetiology, we reported 2 patients with structural aetiology (1 tumour, unknown histology) and 1 with malformation of cortical development (polymicrogyria). Two patients had childhood epilepsy with centro-temporal spikes.

### Patients with known epilepsy or not new-onset seizures

Of 37 patients, 16 (43.2%) were male and 21 (56.8%) female. The median age was 8 years (range 4.00–14.00) [IRQ 6.00–12.00]. Eleven patients (30%) had familiarity for epilepsy and 4 patients (11%) for febrile seizures, and 22 (59%) had not familiarity for epilepsy and/or seizures. In 18 patients (48.6%), neurological clinical examination was normal. Twenty of 37 patients were hospitalized (54%).

At admission, 24 of 37 patients (64.9%) had known epilepsy: 14 (58.3%) were FE, 7 (29.2%) were GE and 3 (12.5%) were UE. In addition, 13 of 37 patients (35.1%) had not known epilepsy: 4 (31%) had FOS, 3 (23%) had GOS and 6 (46%) had unknown onset seizures (UOS).

Thirty-three of 37 patients performed EEG that showed a pathological pattern in 27 (73%) patients: generalized epileptic anomalies in 7 patients and focal anomalies in 13 patients (11 temporal, 5 occipital, 1 frontal, 2 multifocal and 1 hemispheric). Only one patient (3.7%), 9 years old, was photosensitive, with photoparoxysmal response (PPR), manifested by intermittent photic stimulation (IPS).

Thirty-one of 37 patients had undergone CT (11 patients) or MRI (20 patients) at least once in their life. In detail, for 11 patients, MRI (9) and CT (2) showed a structural aetiology: 7 had hypoxic-ischemic syndrome; 2 had a brain tumour (one dysembryoplastic neuroepithelial tumour (DNET), one pilocytic astrocytoma diffuse) and one patient poroencephalic cyst of frontal lobe. Finally, one patient had structural anomalies related to his immune aetiology (Rasmussen’s syndrome).

Discharge diagnoses were epilepsy for 35 of 37 patients (94.6%): 19 (54.3%) were FE; 11 (31.4%) were GE; 2 (5.7%) were combined focal and generalized epilepsy (CFGE) and 3 (8.6%) were unknown epilepsy (UE). On the other hand, 2 of 37 patients (5.4%) had unknown onset seizures.

Regarding epilepsy aetiology, we reported 11 patients with structural aetiology (7 patiens with hypoxic-ischemic syndrome, 2 patients with brain tumour, DNET and PA diffuse, one with poroencephalic cyst, one patient with immune aetiology, Rasmussen’s syndrome). Three patients had a genetic aetiology; they were carrier of mutation in CACNA1A, CUL48 and WDR45 genes. Three patients had childhood epilepsy with centro-temporal spikes.

About patients of previous year, available data were reported in Table 2. Aetiologies of diagnosed epilepsy in all patients were reported in Table 3.

**Table 3:** Epilepsy diagnosis and aetiologies in all population.

Aetiologies	FE	GE	CFGE	UE
Structural	9	-	3	-
Hypoxic-ischemic injury	6	-	1	-
Tumor	2	-	1	-
Malformations of cortical development	-	-	1	-
Porencephalic cyst	1	-	-	-
Genetic	1	2	-	-
Immune	1	-	-	-
Infectious	-	-	-	-
Metabolic	-	-	-	-
Unknown	20*	13	-	3
Total	31	15	3	3

*FE* focal epilepsy, *GE* generalized epilepsy, *CFGE* combined focal and generalized epilepsy, *UE* unknown epilepsy

*5 of them with Childhood epilepsy with centrotemporal spikes.

Nineteen patients were taking antiepileptic drugs, 14 of them (73.7%) had declared a good compliance to the therapy.

## Questionnaire results

Of the 57 enrolled patients, 45 were contacted by phone: 37 of them reported difference in the number of hours of exposure to the devices.

Sixteen of 45 patients (35.5%) had new-onset seizures, while 29 of 45 patients (64.4%) had known epilepsy or not new-onset seizures. In the two different groups, 15 (93.7%) and 27 (93.1%) patients, respectively, confirmed increased use of MMDs during lockdown. Five patients with new onset seizures and 6 with known epilepsy or not new-onset seizures experienced a seizure while using MMDs.

Twenty-one contacted patients (46.6%) reported difference in sleep duration, 6 of them (28.6%) had new-onset seizures. Twenty-three patients (51.1%) referred poor sleep quality, 10 (43.4%) of them had new-onset seizure.

In all our population, there was a statistically significant difference in DST (p = 0.001) and TST (p = 0.045), between period pre- and during lockdown. The median seizure latency from the last use of the device was 3.0 hours (0–24.0) [IQR 0-8.0]. The results were also comparable in the two separate groups. Moreover, only the difference between TST was not significant. Data were reported in Table 4.

**Table 4:** Results of the questionnaire in all contacted patients (45), in patients with new-onset seizures, in patient with known epilepsy or not new-onset seizures.

Daily screen time (DST) and		*p value*
total sleep time (TST)		
All population		
Daily screen time (hours)		
Pre-lockdown	2.5 (0.15–9.0) [IQR 1.5–3.0]	*p* 0.0001
During lockdown	5.8 (2.15–12) [IQR 3.5–8.5]	
Total sleep time (hours)		
Pre-lockdown	9 (7.0–13.5) [IQR 8.0–10.0]	*p* 0.117
During lockdown	8 (6.5–12.0) [IQR 7–10.12]	
Seizure latency	2.75 (0–24) [IQR 0–9.0]	
Patients with new-onset seizure		
Daily screen time (hours)		
Pre-lockdown	2.5 (1.0–5.0) [IQR 1.5–3.0]	*p* 0.0001
During lockdown	7.5 (2.5–12.5) [IQR 6.0–10.0]	
Total sleep time (hours)		
Pre-lockdown	9.0 (7.0–11.0) [IQR 8.0–9.5]	*p* 0.140
During lockdown	8.5 (6.5–9.0) [IQR 7.5–9.0]	
Seizure latency	5 (0–10.0) [IQR 2.0–8.0]	
Patients with known epilepsy or not new-onset seizure		
Daily screen time (hours)		
Pre-lockdown	2.5 (1.0–5.0) [IQR 1.5–3.0]	*p* 0.0001
During lockdown	7.5 (2.5–12.5) [IQR 6.0–10.0]	
Total sleep time (hours)		
Pre-lockdown	9.0 (7.0–11.0) [IQR 8.0–9.5]	*p* 0.140
During lockdown	8.5 (6.5–9.0) [IQR 7.5–9.0]	
Seizure latency	5 (0–10.0) [IQR 2.0–8.0]	

Categorical variables are expressed as a number and percentage. Continuous non-parametric variables are reported as the median, range and interquartile [IQR]. Wilcoxon test was used to compare the hours of DST and TST pre and during lockdown period.

We showed a correlation between DST and age at admission in ED ( Spearman’s *ρ* 0.522, *p* = 0.001) in all included patients. In detail, we confirmed this correlation both in patients with new-onset seizures (Spearman’s ρ 0.573, *p* = 0.025) and in patients with known epilepsy (Spearman’s ρ 0.554, *p* = 0.007).

Considering only patients with a diagnosis of FE and GE, we reported a negative correlation between DST and seizure latency (Spearman’s ρ 0.426, *p* = 0.038). A simple linear regression was calculated to predict interval of time since the last usage based on DST. A significant regression equation was found (F (1.22) = 6.219, *p* = 0.021), with a R^2^ of 0.220. Seizure latency predicted is 10.881–0.861 (DST) hours. Seizure latency decreased 0.861 for each hours of DST. There was no correlation between TST and interval of time since the last usage (p = 0.387). We did not confirm these results analysing data in two separated groups, patients with new-onset seizure [correlation between DST and seizure latency (*p* = 0.384), correlation between TST and seizure latency (p = 0.530)] and patients with known epilepsy (p = 0.244 and *p* = 0.273, respectively).

Seven patients reported the use of one device, 18 patients of two different devices, 13 patients of three, 3 patients of four, 2 patients reported no usage of devices and other 2 patients did not answer this question. There was no difference between the usage of two or more devices and the diagnosis of FE and GE (p= 0.716), neither between the usage of two or more devices and a newly seizure onset (p= 0.821). Predictors for diagnosis of a FE and GE were investigated: no correlation with DST *p* = 0.679, [OR 1.052 (CI 95% 0.826-1.341)], neither regarding TST *p* = 0.065, [OR 1.739 (CI 95% 0.966–3.132)] or the number of used devices *p* = 0.612, OR 1.467 [CI 95% 0.335–6.430] was found. If the analysis was performed in the two separate groups, the results were confirmed as not significant.

## Discussion

During the Covid-19 pandemic, there has been a clinical epidemiological change about the onset of many diseases: in some countries, a decrease of common paediatric diseases has been reported [2, 13], such as asthma and acute respiratory tract infection [14]. Since schools and sport centres closed, the number of acute infections and traumas among children were lower than usual [15]. In addition, the literature reports an increase in sleep disorders, depression and other psychiatric disorders in children, during pandemic [16–19]. Moreover, strong social restrictions allowed children to assume incorrect habits about food, physical activities or screen time [20, 21]. Children, indeed, spent a lot of time at home using MMDs such as TV, smartphones, tablets and video consoles [22]. We showed an increase in the numbers of seizures detected in a paediatric Southern Italian cohort during “quarantine/social limitations” (9th March–4th May 2020), compared with the same population during the same months in 2019. These findings confirm the view that the change in life habits, including the use of mobile devices and sleep changes, might have favoured the occurrence of epileptic seizures in a population of children during the 2020 lockdown.

We witnessed a drastic decrease in admissions in ED in Italy [3] and in Santobono-Pausilipon Children’s Hospital. Also, we reported a significant increase of accesses for seizures during lockdown period.

Normally, the incidence of epilepsy in children ranges from 41 to 187/100000. A higher incidence is reported from underdeveloped countries. The prevalence is consistently higher than incidence and ranges from 3.2 to 5.5/1000 in developed countries and 3.6–44/1000 in undeveloped countries [23–25]. In our study, we analysed the incidence and prevalence of seizures during Italian lockdown for Covid-19 (9th March to 4th May 2020) comparing to the incidence and prevalence of seizures in the same period of last year. We described a significant increase of seizure prevalence and incidence compared with the same period of the last year: the prevalence of seizures in children accessed to the emergency department of Santobono-Pausilipon Hospital in Naples during the lockdown (9th March–4th May 2020) was 2.6%, while in the same period of 2019, the prevalence was 0.4 %, whilst the incidence was 0.94 and 0.13%, respectively, with statistically significant differences in both cases.

These data could reflect a change in the sanitary organization of assistance (reduction of ambulatory activities and migration of patients to the ED access). In our opinion, however, discontinuing of outpatient activities was not very significant. A telemedicine service was created for management and follow-up of known patients, whilst the ambulatory continued to be open for urgency. Most of the patients who benefited from telemedicine were epileptic patients for monitoring and therapeutic modifications. Furthermore, seizures are a reason for access to the emergency department also for the strong emotional impact on parents.

We suspected that some of the conditions experienced during lockdown might have been facilitating factors or triggers for the seizures. In particular, in our study children presented a statistically significant difference in DST and in TST compared with the period before lockdown. In detail, we reported an increasing use of MMDs both in all population and in patients with new-onset seizures or not. We reported, as expected, that older children used devices more than the younger ones. Additionally, considering patients with FE and GE, children that spent more hours using a device, had shorter seizure latency. Precisely, eleven of 45 patients contacted by phone experienced a seizure during device’s use. Furthermore, no gender difference has been described.

It is known that seizures are usually unpredictable and arise spontaneously. Regardless, some perturbations like intercurrent diseases sleep disturbances or emotional stress act as a trigger [26]. In literature seizures (e.g. reflex epilepsy) modulated or precipitated by extrinsic (i.e. light, music) or intrinsic (i.e. movements, emotion, cognitive processes) stimuli are described [26, 27]. In susceptible children, seizures triggered by this type of stimuli or processes can have different latencies [28].

Some studies reported that FE is more frequently related to alterations in the sleep-wake rhythm [29] and GE are frequently triggered by external stimuli [30]. In our study, we noticed no difference in terms of presentation of the seizures between focal and generalized, and no predictive factors (number of hours of sleep and device usage, number of devices used) have been found for FE or GE diagnosis.

In our population, only 2 patients (3.5%) had photosensitivity. In literature, flashing lights are the most frequent extrinsic trigger in reflex epilepsy and occur in 2% of epileptic patients, mainly in adolescents and females. In fact, age-dependent correlation is described [26].

We emphasize that the maximum age to access to our hospital is 14 years. For this reason, data about photosensitivity could be poor. However, reported rate was in line with the literature.

Some authors deny that increased use of MMDs, in particular video games, is related to a higher risk of seizures in non-photosensitive patients. Nevertheless, their studies described adults and only “out-of-date” video games [31–33]. It is known the risk of paroxysmal EEG discharges and consequently of clinical epileptic manifestations for the shortness of the distance between subject and TV screen [33], but we did not investigate it.

Of course, we cannot confirm or not a correlation between seizures and mobile media devices (we have not a control population) but only describe a phenomenon observed during Italian lockdown, in our centre. In our opinion, social restrictions, increased use of devices, sleep deprivation, poor sleep quality and also emotional stress could have been facilitating factors or triggers for seizures. We suggest re-evaluating, with further studies, the role of devices and new games as triggers or facilitating factors of seizures.

As in our population, there was a good compliance to the antiepileptic treatment, and the reported increase of seizures is probably due to a lifestyle variation, with a decrease of sleep time and an increase of device use.

Recently, a noticeable increase in the use of devices, especially in older children, was reported [11, 31]. As announced by American Academy of Pediatrics [34], it is necessary to regulate the use of MMDs, according to the age of the child. It is known, in fact, that overuse of digital media and screens may place children or teenager at risk of obesity, sleep problems, gaming disorder, cyberbullying and risky behaviours (substance abuse, sexual behaviours). In literature the effects of sleep on epileptic seizures are described extensively [35–39]; on the contrary, studies regarding the effects of MMDs on seizures are poor. Probably, seizures might be a possible risk, but future studies are desirable to correlate MMD use with seizures and to understand any pathophysiological mechanisms of them.

Major limitations of the present study are its observational and retrospective design and the lack of a control population to correlate the critical event with the use of devices and the alteration of sleep hours. Moreover, we used only an anamnestic questionnaire, administered via telephone, and the increased number of ED accesses for seizures could reflect the closure of ambulatory facilities in the same period. Finally, we did compare only the same periods of the years 2019–2020, whereas it would have been interesting and more significant to also compare the previous years (at least 5) and to study the trend of accesses during the period pre- and post-lockdown.

## Conclusions

The Covid-19 crisis has imposed numerous social restrictions. Since the pandemic was announced, there has been a high drop of accesses to basic services. Regular playgrounds, social group activities, sports clubs and school have been closed. Social relations have been strongly limited to closest family members. This could harm children and adolescent and change habits and behaviours. In our study we reported an increase of seizures, probably precipitated by higher usage of devices, a reduction in sleep time and impaired sleep quality. Further studies are needed to better characterize the underlying pathophysiological mechanism and the causal link between seizures and devices overuse and sleep disturbances. Moreover, most of the studies are not recent. Maybe, new lifestyles, simultaneous or continuous use of several devices and new games (sometimes violent and stressful) could be considered. Furthermore, recommendations for families and healthcare providers on the use of mobile media by children are strongly needed.

## Electronic supplementary material

ESM 1 (DOCX 15 kb).
