# Supplementary material for: Is Covid-19 lockdown related to an increase of accesses for seizures in the emergency department? An observational analysis of a paediatric cohort in the Southern Italy
Source: Neurol Sci. 2020 Oct 23;41(12):3475–83. doi: 10.1007/s10072-020-04824-5 (PMC7582024; doi:10.1007/s10072-020-04824-5)
Supplement: Supplementary file 1 — (DOCX 15 kb). [file 10072_2020_4824_MOESM1_ESM.docx]

***Appendix 1***

***Devices:***

1. Did your child use electronic devices more during the two weeks before the crisis onset (2 WBCO) compared to an average school period before the lockdown? Y / N

2. How many hours did your child spend (on average) using the devices at that time (the 2WBCO)?

3. How much longer in the 2WBCO than in an average school period before the lockdown, in terms of hours (on average)?

4. How many hours for each of the following devices in the 2 WBCO?

Tv________ Tablet_________ Cell phone__________Computer_______ video console (eg PS, Nintendo, Xbox) _______

5. How much of this time is dedicated to online teaching?

6. How long has it been between the last device’s usage and the onset of the critical episode, in terms of hours?

***Sleep:***

1. Did your child sleep less in the 2 WBCO compared to an average school period before the lockdown? Y / N

2. How many hours did your child sleep (on average, not considering awakenings) in an average school period before the lockdown?

3. How much time did your child sleep in the 2 WBCO compared to an average school period before the lockdown, in terms of hours?

4. Have you noticed any subjective worsening of his/her sleep quality during the lockdown period? Y/N

***Adherence to therapy (if on antiepileptic treatment before the crisis)***

Has your child regularly taken the prescribed antiepileptic drug therapy? Y/N
